# Pascal selective laser trabeculoplasty (PSLT) in the treatment of ocular hypertension and open-angle glaucoma compared to travoprost eye drops: A non-inferiority clinical trial

**DOI:** 10.1371/journal.pone.0350897

**Published:** 2026-07-08

**Authors:** Rafaela C. M. C. de Almeida, Rodrigo L. Lindenmeyer, Fabio Lavinsky, Daniel Lavinsky, Egidio Picetti, Mônica O. da Silva, Diane R. Marinho, Thainá S. Moreira, Victória A. Silveira, Leonardo Leivas, Leonardo da C. Meireles, Helena M. Pakter

**Affiliations:** 1 Ophthalmology, Universidade Federal do Rio Grande do Sul, Porto Alegre, RS, Brazil; 2 Hospital de Clínicas de Porto Alegre, RS, Brazil; 3 Wills Eye Hospital, Philadelphia, Pennsylvania, United States of America; 4 GEPO (Group of Studies and Research in Ophthalmology), Lavinsky Eye Institute, Porto Alegre, Brazil; 5 Ophthalmology, Grupo Hospitalar Conceição, Porto Alegre, RS, Brazil; Daegu Veterans Health Service Medical Center, KOREA, REPUBLIC OF

## Abstract

**Aims:**

To compare the effectiveness of PSLT and the use of topical prostaglandin eye drops in the treatment of ocular hypertensive (OHT) or open-angle glaucoma patients (POAG) in reducing intraocular pressure (IOP) and peak IOP.

**Methods:**

This is a non-inferiority randomized clinical trial, after washout, both patients eyes were randomized, one eye treated with PSLT (intervention group) and the contralateral eye treated with travoprost eye drops (controls). IOP, peak IOP by water drinking test (WDT), visual field (VF) and OCT were performed at baseline and at 7 days, 2, 6, 9 and 12 months after treatment. The primary outcome measure was to assess if the impact of PSLT on IOP and peak IOP is not inferior to that of the standard treatment (travoprost eye drops) by a margin not greater than the predefined threshold of 1.5 mmHg.

**Results:**

A total of 30 patients participated in this trial. The mean baseline IOP was similar in both groups, 18.8 ± 4.0 mmHg and 18.8 ± 4.2 mmHg, intervention and control respectively. At 2, 6 and 9 months of treatment no significant difference in IOP was found between the two groups. At 12 months of follow-up the PSLT group showed lower average IOP (13.5 ± 2.3 mmHg versus 14.9 ± 2.7 and p < 0.001). Similar results were found for peak IOP in the WDT. On average there was no progression over time and no significant difference between groups regarding VF and OCT parameters.

**Conclusion:**

PSLT was as effective as prostaglandin eye drops in reducing IOP and IOP peak in POAG and OHT patients. These findings are especially important as interest grows in minimally invasive glaucoma treatments and reducing reliance on topical medications.

**Trial registration:**

Clinicaltrials.gov NCT05241938

## Introduction

Glaucoma affects approximately 80 million people worldwide and is a leading cause of irreversible blindness, with a significant social and economic impact [1]. In Brazil, there is a lack of information regarding the prevalence of glaucoma. Most studies are restricted, outdated and show a prevalence of 2% − 3% in the population over 40 years old, with an increase in prevalence as age increases [2,3].

Topical pharmacological therapy continues to be the main strategy of reducing IOP used in the management of ocular hypertension and glaucoma, being prostaglandin analog eye drops first line therapy. The disadvantages of using eye drops include intolerance, the need for daily application, costs and patient compliance [4,5].

Selective Laser Trabeculoplasty (SLT) has currently become an important therapeutic option for treating glaucoma. After the publication of the Laser in Glaucoma and Ocular Hypertension (LiGHT) [6] study in 2019, changes have been noted in clinical practice. That multicenter, randomized clinical trial concluded that initiating treatment with SLT is cost-effective, with no significant difference in quality of life, as compared to conventional eye drops treatment as the first choice [6].

A computer-guided pattern scanning laser, which has been used for focal or panretinal photocoagulation (PRP), can also be used for trabeculoplasty in glaucoma patients. The first manufacturer of Pascal selective laser trabeculoplasty (PSLT) (Pascal Streamline 577, Topcon, Inc) received authorization for clinical use in the treatment of ocular hypertensive and glaucomatous patients in 2018. This technique ensures complete trabecular meshwork treatment coverage and proper alignment of the shot sequence, despite the absence of visible burns via a treatment algorithm and no significant overlaps or gaps [7]. The advantages of PSLT over SLT are increased precision and accurate application, reduced procedure time due to automation, and less thermal damage which minimizes collateral tissue damage. There are still few studies with PLST, four previous studies evaluated the safety and efficacy of PSLT in eyes with glaucoma (Turati et al [7], Barbu et al [8], Al Zubi et al [9] and Espinoza et al [10]) and three randomized clinical trials compared the two treatment modalities with selective trabeculoplasty to laser (SLT and PSLT) (Kaweh Mansouri et al [11], Elahi S et al [12], Wong MOM et al [13]). These studies found no significant differences between SLT and PSLT in terms of efficacy and safety in treating patients with glaucoma and achieved success rates in reducing IOP in both procedures.

Based on our current understanding, this trial marks the first attempt to compare the effectiveness of PSLT and travoprost eye drops in reducing intraocular pressure (IOP) and peak IOP in patients with POAG or OHT. Since the Pascal laser combines both retina and glaucoma modules, it might provide versatile treatment options for a wider array of ophthalmic conditions in one device and could have a greater impact on healthcare.

## Materials and methods

### Design

This study was a prospective non-inferiority randomized clinical trial (RCT). Patients who consulted consecutively at the ophthalmology service of the Hospital de Clínicas de Porto Alegre (HCPA), and who were diagnosed with bilateral ocular hypertension (OHT) or primary open angle glaucoma (POAG), and using up to two classes of hypotensive eye drops were invited to participate. The study protocol adhered to the principles of Helsinki declaration, was approved by the hospital research ethics committee (CAAE: 44489320.7.0000.5327), registered at clinicaltrials.gov (NTC 05241938), and followed the Consolidated Standards of Reporting Trials (CONSORT) guidelines [14]. Patients who agreed to participate and signed an informed consent form were included in the study, and data from both eyes were collected. Recruitment and baseline were conducted from September 1, 2021 to July 25, 2022 and the 12-month follow-up was conducted from September 1, 2022 to July 26, 2023.

The lead investigators and examiners in the trial were glaucoma-trained specialists.

### Patients

Patients were included in the study if they were 20 years old or older with OHT or POAG with 360° open iridocorneal angles in both eyes. The definition of OHT was considered IOP ≥ 24 mmHg with central corneal thickness < 600 µm, open angle on gonioscopy, absence of optic nerve abnormalities, consistent with glaucoma, and absence of abnormalities in two white-on-white standard automated visual fields (VF). Glaucoma diagnosis required the presence of glaucomatous optic neuropathy, including, but not restricted to, loss of the neuroretinal rim, increased size and depth of the optic nerve excavation, nerve fiber layer thinning, visual field outside normal limits (as verified by the glaucoma hemifield test), and/or a cluster of three or more non-edge points in a location typical for glaucoma, with all points depressed on the pattern deviation plot at the p < 5% level and at least one point depressed at the p < 1% level.

The inclusion criteria were: visual acuity 20/40 or better, spherical equivalent within 6.0 D to −6.0 D, and no major ocular trauma or ocular surgeries other than uncomplicated cataract. The exclusion criteria were: unilateral glaucoma, use of more than two glaucoma medications, evidence of other ocular diseases that could affect the studied parameters (e.g., presence diabetic or hypertensive retinopathy), use of medications known to increase IOP (i.e., corticosteroids), diagnosis of other types of glaucoma (e.g., secondary glaucoma, congenital glaucoma), or advanced glaucoma (defined as visual field MD of less than −12 dB and/or a vertical cup-disc ratio of 0.9 or greater).

### Visits an examination

At baseline, detailed demographic characteristics and medical history were collected using a standardized questionnaire and all patients underwent a complete ophthalmic examination, including visual acuity, refraction, slit lamp biomicroscopy, gonioscopy, mydriatic fundus assessment and central corneal thickness measurement (Ocuscan RXP Pachymeter, Alcon, USA).

After 4-week washout in both eyes, randomization was performed (using random.org), to allocate one eye of each patient to receive PSLT (intervention group), while the fellow eye received a prescription for travoprost 0.04% prostaglandin analog eye drops (control group).

Intraocular pressure was measured in every visit, from 8:00 am to 10:00 am, by one examiner masked to treatment randomization, using applanation tonometry (Perkins tonometer, Haag-Streit, UK). To assess IOP peak, a water drinking test (WDT) was performed. The test consists of a baseline IOP measurement, followed by measurements at 15, 30, and 45 minutes after drinking 1 liter of water in less than 5 minutes. For this test, patients were fasting for 2 hours. The baseline IOP was the IOP measured before the ingestion of water, and the IOP peak was determined as the highest IOP measured during the WDT.

Standard white-on-white computerized visual field 24−2 (Humphrey Field Analyzer 740i, Carl Zeiss Meditec, Swedish) was used. The reliability indices were set at false-positive rates ≤10%, as well as false-negative rates and fixation losses ≤15% on at least two consecutive examinations.

The patients also underwent swept source optical coherence tomography (SS-OCT Triton, Topcon, Japan). A 12 × 9 mm wide-field cube was routinely performed, including analysis of the circumpapillary retinal nerve fiber layer (cpRNFL), macular RNFL and ganglion cell-inner plexiform layer (GCL++). The scans qualified if they presented a quality index >40, with no motion artifacts determined by the discontinuity of the major blood vessels that exceeded the width of 1 major vessel diameter, no decentration of the peripapillary circle, nor segmentation defects.

In the intervention group, computer-guided PSLT was performed by a single ophthalmologist using a Pascal Streamline 577 laser (Topcon Inc., Tokyo, Japan), under topical anesthesia (5 mg/ml proxymetacaine hydrochloride eye drops), using a mirrored gonioscopy lens. Brimonidine Tartrate 0.2% and pilocarpine 2% eye drops were applied 40 minutes before the laser treatment. The laser spot was directed at the angle of the anterior chamber covering the pigmented and non-pigmented trabecular meshwork. Laser power was titrated by positioning a laser mark in the lower quadrant with a 10 ms exposure duration. The initial energy of 500 mW was selected, and the power was reduced or increased until a slight whitening of the trabecular meshwork was minimally noticed. This power was then maintained, and the pulse duration automatically reduced to 5 ms to produce no visible signs of tissue injury. The treatment was administered in 32 steps, each pattern consisting of 36 points: three rows of 13 points each (total of 1152), with zero space between adjacent points. After laser treatment, the patient was instructed to use non-steroidal anti-inflammatory eye drops (TID for seven days). In the control group, travoprost 0.04% eye drops were prescribed to be used continuously, applying one drop at night, in the contralateral eye to the PSLT. Treatment modification was permitted if a progression in VF and/or OCT was noted at any follow-up visit.

The measurements were conducted at baseline and at the following intervals: IOP was recorded at 1 week (to evaluate the initial IOP response and early safety after PSLT, as laser trabeculoplasty procedures are known to produce early changes in IOP and, in some cases, transient IOP fluctuations shortly after treatment) and 2, 6, and 12 months (to assess the stabilization of the treatment effect and to allow sufficient time for the full IOP-lowering effect of trabeculoplasty to develop). WDT IOP at 2 and 12 months; VF examinations at 6 and 12 months; and OCT scans at 12 months. A single examiner performed all IOP measurements, a second examiner performed the VF, and a third examiner performed the OCT. Importantly, all examiners were masked to the treatment allocation.

### Outcomes measures

The primary outcome measure was to assess if the impact of PSLT on IOP is not inferior to that of the standard treatment by a margin not greater than the predefined threshold of 1.5 mmHg. The secondary outcome measures were reduction of IOP peak in WDT, and absence of progression in VF and OCT examinations.

### Statistical analysis

Study data were collected and managed using REDCap electronic data capture tools hosted at Yale University. The SPSS Statistics 11.0 software was used to analyze data. Continuous variables that follow a normal distribution are expressed as mean ± standard deviation (SD). Categorical variables are described in terms of frequencies and percentages with 95% confidence intervals (CI). Analysis of comparative data was performed using generalized estimating equations (GEE) with normal distribution, identity link and exchangeable correlation matrix considering that eyes are nested for each patient. Statistical significance was defined at p < 0.05. A sample size of 56 eyes (28 per group) was estimated to test the non-inferiority of 1.5 mmHg in terms of mean IOP, comparing intervention to control group. Power was set at 80%, with significance level of 5%, and SD equal to 3.5 mmHg. This estimation was obtained using the Power and Sample Size (PSS) for Health Researchers tool. The non-inferiority margin of 1.5 mmHg was based on clinical reviews indicating that IOP measurement differences of up to 2 mmHg are likely the result of measurement variability or error. From a clinical perspective, differences in mean IOP reduction of approximately 1–2 mmHg are generally considered small in comparative studies of treatments for ocular hypertension and primary open-angle glaucoma [15,16].

## Results

**Fig 1:**
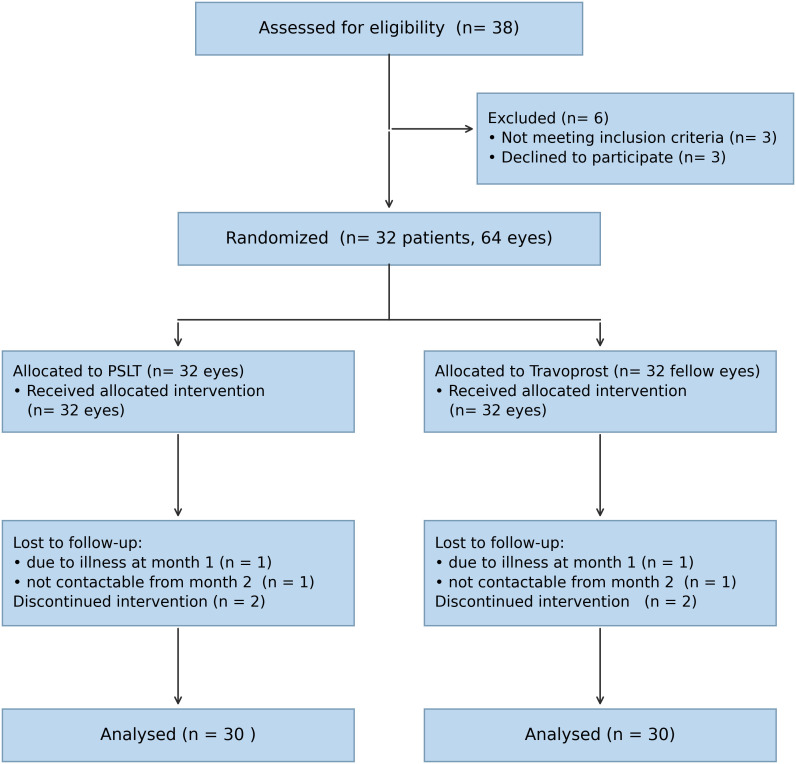
Screening, Recruitment, and randomization CONSORT Flow Diagram.

A total of 38 patients were assessed for eligibility; 6 were excluded. Thirty-two patients (64 eyes) were randomized, and 30 completed follow-up and were included in the final analysis. Most patients were female (76.7%) and mean age was 60.9 ± 11.7 years (range 26–83). Table 1 depicts subject’s baseline characteristics and a flow diagram (Fig 1) provides a comprehensive visual summary of the progress of all participants through the phases of this RCT.

**Table 1:** Patients’ baseline characteristics.

	Percentage
Female Sex	76.7
White Race	60
Black Race	26.7
Other Race	13.3
Positive Family History	50
Systemic Hypertension	70
Diabetes	40
Pseudophakic	6.6
	Mean ± SD
Age	60.9 ± 11.7
CCT PSLT	537.1 ± 33.4*
CCT Eye drops	539.5 ± 36.9
Baseline VF MD PSLT	−3.6 ± 3.1*
Baseline VF MD Eye drops	−4.3 ± 3.2

SD – Standard Deviation

CCT – Central Corneal Thickness

VF MD – Visual Field Mean Deviation

*No significant difference between groups

Baseline mean IOP was similar in both groups (intervention 18.8 ± 4.0 mmHg and control 18.8 ± 4.2 mmHg, p > 0.05). At one week follow-up, mean IOP decrease was greater in the control group (mean IOP 16.8 ± 4.2 mmHg and 14.8 ± 3.1 mmHg, intervention and control group respectively, p < 0.001). At two, six, and nine months post-treatment, we found no significant difference in IOP between the two groups. At twelve months follow-up, mean IOP decrease was greater in the intervention group (13.5 ± 2.3 mmHg and 14.9 ± 2.7, intervention and control group group respectively, p < 0.001). Table 2 shows the mean IOP values for each evaluated month. We noted that the intervention group showed a constant decrease in IOP over time, whereas the control group showed stable IOP. There was a significant reduction in WDT peak IOP at 2 months of follow-up in both groups, with no significant difference in effect between the groups (baseline vs 2 months follow-up mean peak IOP in intervention group 23.3 ± 4.6 mmHg to 18.9 ± 4.3 mmHg, and control group 23.6 ± 4.8 to 18.6 ± 4.3 mmHg, p > 0.05). At 12 months follow-up, mean peak IOP in the intervention group was significantly lower than the control group (15.5 ± 2.4 mmHg to 17.8 ± 2.6 mmHg, respectively, p < 0.001) shown at Table 2.

**Table 2:** Mean IOP and WDT IOP peaks, in mmHg ± SD.

Treatment	Moment	Mean	95% Wald Confidence interval	
			Lower	Upper
Average IOP (±SD, mmHg)				
Intervention: PSLT	Baseline	18,8 ± 4,0	17,3	20,2
	1 week	16,8 ± 4,2	15,3	18,3
	2 months	15,7 ± 3,3*	14,5	16,8
	6 months	14,7 ± 3,5	13,4	15,9
	9 months	13,9 ± 2,8	12,9	14,9
	12 months	13,5 ± 2,3*	12,6	14,3
Control: Eye Drops	Baseline	18,8 ± 4,2	17,3	20,2
	1 week	14,8 ± 3,1*	13,7	15,9
	2 months	15,0 ± 3,9	13,6	16,4
	6 months	14,8 ± 3,7	13,5	16,1
	9 months	14,3 ± 3,2	13,2	15,4
	12 months	14,9 ± 2,7*	13,9	15,8
IOP peaks during water drinking test (±SD, mmHg)				
Intervention: PSLT	Baseline	23,3 ± 4,6	21,7	25
	2 months	18,9 ± 4,3	17,4	20,4
	12 months	15,5 ± 2,4	14,7	16,4
Control: Eye Drops	Baseline	23,6 ± 4,8	21,9	25,4
	2 months	18,6 ± 4,3	17,1	20,2
	12 months	17,8 ± 2,6	16,9	18,7

p < 0.001 for interaction between group and time of the generalized estimating equations (GEE) model

* p < 0.001 from multiple comparisons test with Bonferroni adjustment

IOP – Intraocular Pressure WDT – water drinking test SD – Standard Deviation

**Fig 2:**
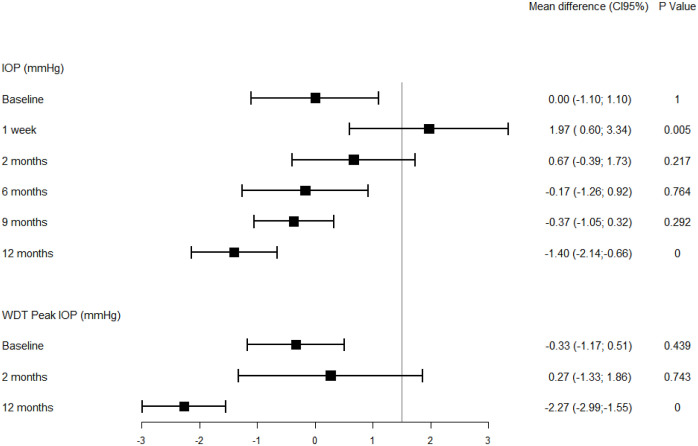
Forest plot illustrating the mean difference in intraocular pressure (IOP) and peak IOP (WDT) among treated groups. Each point estimate denotes the mean difference in IOP between PSLT and Travoprost eye drops (squares), with accompanying 95% confidence intervals (whiskers). The vertical line denotes the non-inferiority margin at 1.5 mmHg.

The non inferiority of the PSLT is shown in Fig 2, a forest plot for the mean difference between groups in IOP and peak IOP (WDT). It demonstrates that PSLT is not significantly worse than travoprost eye drops by more the maximum acceptable difference in IOP which was set at 1.5 mmHg (vertical line). In fact, at the 12 months follow up, PSLT was significantly superior.

Regarding functional and structural changes, no progression was detected in any group, this was already expected due to the short duration of the study.

The most common adverse events were conjunctival hyperemia and keratitis in eyes treated with travoprost eye drops, but it was not necessary to suspend or replace the medication in any patient. Only one patient required additional therapy, 0.2% brimonidine eye drops during 1 month, due to a hypertensive peak after the PSLT. Another patient presented a small hyphema after laser treatment with spontaneous resolution. A third patient reported irregular use of the eye drops, whereas two patients discontinued the use of the eye drops on their own, after 10 and 11 months of follow-up each.

## Discussion

To the best of our knowledge, this is the first non-inferiority clinical trial to compare the effectiveness of PSLT and the use of travoprost eye drops in lowering IOP and IOP peak in POAG or OHT patients.

A non-inferiority margin (Δ) of 1.5 mmHg was prespecified for the difference in mean intraocular pressure (IOP) between treatments, defined as intervention minus control. At 1 week, the mean IOP in the intervention group remained higher than the control group, and the observed difference exceeded the predefined margin. Therefore, non-inferiority of PSLT was not demonstrated at this early time point. The lack of non-inferiority at this visit is consistent with the known temporal profile of selective laser trabeculoplasty, as the IOP-lowering effect of the procedure typically develops over several weeks, whereas topical medications produce an immediate pharmacologic effect.

At subsequent follow-up visits (2, 6, and 9 months), the difference in mean IOP between treatments decreased substantially and remained small, suggesting comparable IOP control between intervention and control groups during the follow-up period.

Although the study was designed as a non-inferiority trial, the results at 12 months indicate that PSLT achieved a greater IOP reduction than topical therapy. In the present analysis, the mean reduction in IOP was greater in the intervention group (mean difference −1.4 mmHg; 95% CI, −2.65 to −0.15; p = 0.030). As the entire confidence interval lies below both the non-inferiority margin and zero, favoring PSLT, these findings indicate that PSLT not only met the criterion for non-inferiority but also demonstrated statistical superiority over topical therapy. The significant difference in mean IOP between treatments, which favors the intervention group at the end of follow-up, could also be related to poor adherence to topical treatment. The insufficient adherence to therapy among glaucoma patients is a recurrent issue. The ophthalmological literature shows low rates of treatment adherence (30–70% of prescribed medication doses), which is associated with disease progression and increased complication rates, as well as healthcare costs [17,18].

The mechanisms of PSLT to reduce IOP are not yet known and longer follow-up is needed to assess the longevity of its IOP-lowering effect and whether it can be repeated. SLT presents a rich literature; however, studies on PSLT show a follow-up time up to 18 months and none discuss the possibility of repeating the treatment. The LiGHT study has followed patients for six years and demonstrated that SLT allowed successful drop-free IOP control in almost 70% of eyes after that period [19]. Regarding laser repeatability, studies consistently show that SLT can be reapplied, with pressure success rates and duration similar to the initial procedure [20]. It is reasonable to assume that PSLT performs similarly to SLT in those matters, but longer studies are needed to verify this hypothesis.

In this sample, there was not a cut off limit for baseline IOP after washout, and thus five patients with PIO ≤ 21 mmHg were included. Also the mean IOP after washout was close to 18 mmHg, in both groups. Considering that pre-treatment IOP is the main predictor of success after SLT, this could have interfered with the magnitude of our results. Eyes with higher pre-treatment IOP tend to have a greater drop in pressure, but patients with normal pressure glaucoma also benefit from SLT. Although this type of glaucoma shows a lower baseline IOP and consequently less IOP reduction, the laser can reduce daytime IOP fluctuations, thus stabilizing a worsening of the visual field without necessarily manifesting a lower IOP [6,21–24].

This study shows potential limitations. Patients could be on treatment with any hypotensive eye drops before enrolled in the study and, despite conducting a washout period, this could have influenced the response to the laser treatment, especially in those previously using prostaglandins analogs. Considering that prostaglandins and SLT present similar mechanisms of action, it is hypothesized that prior therapy with this class of medications may affect the subsequent efficacy of SLT [18, 25].

In addition, since both eyes of the same patient were included in the study, it is possible that there was a crossover effect from one eye to the other [26]. SLT appears to hold a statistically significant pressure-lowering effect in the untreated eye in some studies. A reduction of 8–12% in IOP in the contralateral eye has been reported after 6–12 months of treatment with SLT in some studies [21,27–29]. The potential crossover effect of PSLT only makes our results stronger because even with this pressure-lowering effect in the untreated eye, drops were less effective. On the other hand, it is also possible that prostaglandin eye drops could cause a decrease in IOP in the contralateral eye, but the literature is controversial in this regard.

Considering that pre-treatment IOP is the main predictor of success after SLT and our relatively small sample size did not allow for a stratified analysis of OHT versus POAG patients some patients had lower baseline IOP, which may also interfere with the results in both groups [6,21–24].

We conclude that PSLT demonstrates comparable efficacy to travoprost eye drops in treating glaucoma and OHT patients. Our findings indicate that PSLT is also safe and, as well as SLT, could be considered as the primary therapeutic option. Given that the Pascal laser integrates both retina and glaucoma modules, it offers therapeutic versatility for a broader range of ophthalmic conditions within a single device. Consequently, it may exert a more significant influence on healthcare expenditures.
